# A pilot study to evaluate tissue- and plasma-based DNA driver mutations in a cohort of patients with pancreatic intraductal papillary mucinous neoplasms

**DOI:** 10.1093/g3journal/jkac314

**Published:** 2022-12-01

**Authors:** Margaret A Park, Thinzar Zaw, Sean J Yoder, Maria Gomez, Maria Genilo-Delgado, Toni Basinski, Esther Katende, Aamir Dam, Shaffer R S Mok, Alvaro Monteiro, Amir Mohammadi, Daniel K Jeong, Kun Jiang, Barbara A Centeno, Pamela Hodul, Mokenge Malafa, Jason Fleming, Dung-Tsa Chen, Qianxing Mo, Jamie K Teer, Jennifer B Permuth

**Affiliations:** Department of Gastrointestinal Oncology, H. Lee Moffitt Cancer Center & Research Institute, Tampa, FL 33620, USA; Department of Biostatistics and Bioinformatics, H. Lee Moffitt Cancer & Research Institute, Tampa, FL 33620, USA; Department of Gastrointestinal Oncology, H. Lee Moffitt Cancer Center & Research Institute, Tampa, FL 33620, USA; Molecular Genomics Core Facility, H. Lee Moffitt Cancer Center & Research Institute, Tampa, FL 33620, USA; Department of Gastrointestinal Oncology, H. Lee Moffitt Cancer Center & Research Institute, Tampa, FL 33620, USA; Department of Gastrointestinal Oncology, H. Lee Moffitt Cancer Center & Research Institute, Tampa, FL 33620, USA; Department of Gastrointestinal Oncology, H. Lee Moffitt Cancer Center & Research Institute, Tampa, FL 33620, USA; Department of Gastrointestinal Oncology, H. Lee Moffitt Cancer Center & Research Institute, Tampa, FL 33620, USA; Department of Gastrointestinal Oncology, H. Lee Moffitt Cancer Center & Research Institute, Tampa, FL 33620, USA; Department of Gastrointestinal Oncology, H. Lee Moffitt Cancer Center & Research Institute, Tampa, FL 33620, USA; Department of Cancer Epidemiology, H. Lee Moffitt Cancer Center & Research Institute, Tampa, FL 33620, USA; Department of Gastrointestinal Oncology, H. Lee Moffitt Cancer Center & Research Institute, Tampa, FL 33620, USA; Department of Diagnostic Imaging and Interventional Radiology, H. Lee Moffitt Cancer Center & Research Institute, Tampa, FL 33620, USA; Department of Anatomic Pathology, H. Lee Moffitt Cancer & Research Institute, Tampa, FL 33620, USA; Department of Anatomic Pathology, H. Lee Moffitt Cancer & Research Institute, Tampa, FL 33620, USA; Department of Gastrointestinal Oncology, H. Lee Moffitt Cancer Center & Research Institute, Tampa, FL 33620, USA; Department of Gastrointestinal Oncology, H. Lee Moffitt Cancer Center & Research Institute, Tampa, FL 33620, USA; Department of Gastrointestinal Oncology, H. Lee Moffitt Cancer Center & Research Institute, Tampa, FL 33620, USA; Department of Biostatistics and Bioinformatics, H. Lee Moffitt Cancer & Research Institute, Tampa, FL 33620, USA; Department of Biostatistics and Bioinformatics, H. Lee Moffitt Cancer & Research Institute, Tampa, FL 33620, USA; Department of Biostatistics and Bioinformatics, H. Lee Moffitt Cancer & Research Institute, Tampa, FL 33620, USA; Department of Gastrointestinal Oncology, H. Lee Moffitt Cancer Center & Research Institute, Tampa, FL 33620, USA; Department of Cancer Epidemiology, H. Lee Moffitt Cancer Center & Research Institute, Tampa, FL 33620, USA

**Keywords:** circulating DNA, somatic mutations, pancreatic cysts, biomarkers, pancreatic cancer, early detection

## Abstract

Intraductal papillary mucinous neoplasms (IPMNs) are precursor lesions to pancreatic ductal adenocarcinoma that are challenging to manage due to limited imaging, cytologic, and molecular markers that accurately classify lesions, grade of dysplasia, or focus of invasion preoperatively. The objective of this pilot study was to determine the frequency and type of DNA mutations in a cohort of surgically resected, pathologically confirmed IPMN, and to determine if concordant mutations are detectable in paired pretreatment plasma samples. Formalin-fixed paraffin-embedded (FFPE) tissue from 46 surgically resected IPMNs (31 low-grade, 15 high-grade) and paired plasma from a subset of 15 IPMN cases (10 low-grade, 5 high-grade) were subjected to targeted mutation analysis using a QIAseq Targeted DNA Custom Panel. Common driver mutations were detected in FFPE from 44 of 46 (95.6%) IPMN cases spanning all grades; the most common DNA mutations included: *KRAS* (80%), *RNF43* (24%), and *GNAS* (43%). Of note, we observed a significant increase in the frequency of *RNF43* mutations from low-grade to high-grade IPMNs associated or concomitant with invasive carcinoma (trend test, *P* = 0.01). Among the subset of cases with paired plasma, driver mutations identified in the IPMNs were not detected in circulation. Overall, our results indicate that mutational burden for IPMNs is a common occurrence, even in low-grade IPMNs. Furthermore, although blood-based biopsies are an attractive, noninvasive method for detecting somatic DNA mutations, the QIAseq panel was not sensitive enough to detect driver mutations that existed in IPMN tissue using paired plasma in the volume we were able to retrieve for this retrospective study.

## Introduction

In the United States, pancreatic ductal adenocarcinoma (PDAC) is the third leading cause of cancer deaths with a 5-year relative survival rate of 11% (Heitzer *et al*. 2019; Siegel *et al*. 2021). To reduce morbidity and mortality, an emphasis on early detection and prevention strategies is greatly needed (Heitzer *et al*. 2019). Intraductal papillary mucinous neoplasms (IPMNs) are common cystic precursor lesions to PDAC that account for nearly half of the asymptomatic pancreatic cysts detected incidentally by imaging each year (Chadwick *et al*. 2009).

IPMNs range in severity from low/moderate-grade lesions that are frequently surveyed to high-grade and invasive lesions that warrant resection. Risk stratification continues to be challenging and often determined by antiquated clinical guidelines (Tanaka *et al*. 2017). The development of minimally invasive biomarkers would greatly assist in the proper characterization and management (Sachs *et al*. 2009).

Next-generation sequencing (NGS) has been used to detect somatic mutations which may classify pancreatic cysts and their grade of dysplasia (Amato *et al*. 2014; Simen *et al*. 2015; Chae *et al*. 2017; Singhi *et al*. 2018). It has been established that several activating mutations such as *KRAS* and *GNAS* and inactivating mutations in *RNF43* are commonly detected in IPMNs, with *GNAS* and *KRAS* mutations allowing discrimination between mucinous and nonmucinous cysts (Sato *et al*. 2001; Biankin *et al*. 2002; Schönleben *et al.* 2006; Abe *et al*. 2007; Schönleben *et al*. 2009). Less commonly detected mutations are found in *BRAF*, *PIK3CA*, *STK11*, and *SMAD4* (Wu, Jiao, *et al.* 2011; Wu, Matthaei, *et al.* 2011; Yamaguchi *et al*. 2011). Interestingly, mutations in *RNF43*, *TP53*, *PIK3CA*, *PTEN*, and/or *AKT1* have more frequently been found in higher-grade rather than low- or moderate-grade IPMNs (Schönleben *et al*. 2009; Amato *et al*. 2014; Singhi *et al*. 2018). While NGS has typically been used to analyze gene mutations in resected pancreas tumor samples, studies have also shown the diagnostic performance of NGS in pancreatic cyst fluid (PCF; Singhi *et al*. 2018).

In a small study of PCF samples from 19 patients, the same *KRAS* and *GNAS* mutations were identified in PCF and its corresponding cystwalls (Wu, Matthaei, *et al.* 2011). In another study of PCF from 10 intestinal-type IPMNs, sequencing detected 10 of 13 mutations present in matched IPMN tissue, including 6 of 7 *GNAS* mutations and each of 3 *KRAS* mutations (Berger *et al*. 2016). Finally, results of one large, prospective study of endoscopic ultrasound-fine needle aspirate (EUS-FNA) obtained preoperative PCF showed high sensitivity (89%) and specificity (100%) in classifying pancreatic cysts using *KRAS* and *GNAS* mutation status (Singhi *et al*. 2018). Although PCF may serve as an important reservoir for biomarkers, the process of EUS-FNA poses risks such as bleeding, infection, and pancreatitis. As such, obtaining biospecimens by less invasive means is of paramount interest.

Circulating tumor DNA from blood is emerging as a diagnostic and prognostic biomarker for patients with PDAC, with existing studies focusing on the detection of *KRAS* and/or *GNAS* mutations. For example, a study conducted by Pietrasz and colleagues showed that *KRAS* mutations detected in plasma by NGS and droplet-based digital PCR can improve the prognostic staging of metastatic and locally advanced PDAC (Pietrasz *et al*. 2017). In another study of circulating cell-free DNA (cfDNA) isolated from whole blood, cfDNA analyzed for *GNAS* codon 201 mutations and *KRAS* codon 12 mutations discriminated between individuals without pancreatic lesions and patients with branch-duct IPMNs or PDAC with 81% accuracy (Berger *et al*. 2016). Finally, Hata *et al*. (2020) demonstrated that the prevalence of *GNAS* mutations in cfDNA of patients with IPMNs was significantly higher than other pancreatic cystic neoplasms (Hata *et al*. 2020).

The goal of this pilot study was to conduct the first investigation to assess whether somatic mutations present in surgically resected, pathologically confirmed pancreatic IPMN cases can also be detected in paired pretreatment plasma samples by conducting NGS for a customized panel of 22 genes reported to play a role in pancreatic carcinogenesis.

## Methods

### Patient cohort

In this proof-of-concept study, we retrospectively reviewed data from a cohort of surgically resected, pathologically confirmed IPMN cases between 2006 and 2011 at Moffitt Cancer Center (Tampa, FL, USA). Patients were consented for specimens to be donated for research through an institutional banking protocol known as total cancer care (Fenstermacher *et al*. 2011). The diagnosis and grade of dysplasia for each IPMN were pathologically confirmed using the 4-tier World Health Organization (WHO) classification guidelines available at the time of diagnosis (i.e. low-grade, moderate-grade, high-grade, and invasive IPMN; Flejou 2011; Nagtegaal *et al*. 2020). For the purposes of this study and to update the classification of IPMNs, an additional review of the medical records was performed and a new two-tier classification (low-grade, high-grade) under the 2019 WHO guidelines was made using clinical and pathological features in the pathology reports (Nagtegaal *et al*. 2020). Informed consent was obtained under an institutional review board-approved protocol and documented in the electronic medical record.

### Sample processing, DNA isolation, and quality control (QC)

To be eligible for this study, available clinical data, 46 formalin-fixed paraffin-embedded (FFPE) tissue blocks, and matched preoperative plasma (for a cohort of 15 patients) were required. Tissues were obtained from the surgical suite, harvested, and reviewed by a pancreatic pathologist (KJ), to verify the diagnosis. Blood samples were collected from consented participants via phlebotomy in a 10-mL EDTA tube and processed for plasma within 2 h using standard procedures, aliquoted into 0.5-mL bar-coded cryovials, and banked at −80°C. None of the cases received chemotherapy or radiation prior to resection. For this retrospective study, both FFPE and plasma samples were identified and retrieved by the Moffitt Cancer Center Tissue Core Facility.

#### FFPE

A total of 46 resected IPMN samples from unique patients were analyzed. Tissue from the cystic wall with the highest grade of dysplasia was obtained from donor blocks using 1 mm core punches. The tissue cores from identified cases underwent DNA extraction using the QIAamp DNA FFPE Kit according to manufacturer's instructions. DNA was qPCR-quantitated using the Kapa Library Quantification Kit (Roche, Inc. New York, NY, USA) and underwent electrophoretic QC using a bioanalyzer and established procedures (Simen *et al*. 2015).

#### Plasma

A total of 17 cases were identified with patient-matched plasma from our original cohort of 46 patients. For this subset, Moffitt Cancer Center's Tissue Core retrieved and thawed 1–0.5 mL cryovial of plasma. Plasma cases underwent DNA extraction using the QIAamp MinElute ccfDNA kit according to manufacturer's instructions. DNA was qPCR-quantitated as for FFPE and underwent electrophoretic QC using a bioanalyzer and established procedures (Simen *et al*. 2015). Samples with contaminating cellular DNA (determined via BioAnalyzer) were excluded from the study (*n* = 2).

### Mutation analysis

Genes were targeted using a QIAseq Custom Gene Panel (Cat. No CDHS-16158Z-1042, Product no. 333525), which covered various hotspot mutations and exons in genes implicated in pancreatic carcinogenesis: *AKT1*, *APC*, *ATM, BRAF*, *BRCA1*, *BRCA2*, *CDKN2A*, *CTNNB1*, *GNAS*, *KRAS*, *MLH1*, *MSH2*, *MSH6*, *PALB2*, *PIK3CA*, *PMS1*, *PMS2*, *PTEN*, *RNF43*, *SMAD4*, *STK11*, and *TP53*.

Capture libraries were sequenced with a 2 × 150 base paired-end sequencing run on the Illumina NextSeq 500. Sequence analysis and mutation detection were performed using the QIAGEN Genomics Workbench (Qiagen, Germantown, MD, USA) and smCounter2 (Xu *et al*. 2019). VarSifter was used to compare identified mutations to the 1,000 genomes project (Teer *et al*. 2012). A cutoff frequency of 2% was used for alternative allele frequency, then known driver mutations in the genes listed above were manually curated using COSMIC as a guide to prevent the identification of germline SNPs in paired and unpaired samples. Only protein-coding variants and variants in the 2 nucleotides immediately preceding and following exons were considered in this study.

### Statistical analysis

Frequencies of known driver mutations detected were evaluated in each targeted gene. Cases were classified according to IPMN grade using past 4-tier^27^ or present two-tier WHO criteria (Flejou 2011; Nagtegaal *et al*. 2020). Association of grade and frequency of mutations (mutation yes/no) was examined by the chi-square test. The trend of mutation frequency by grade was examined by a Cochran–Armitage test. For a subset of patients (*n* = 15), plasma DNA (ptDNA) was compared to specimen DNA (tDNA) to assess concordance for known driver mutational status. *P*-values are two-sided, and a *P*-value <0.05 was considered statistically significant.

## Results

### Select characteristics of the study population

A total of 46 patients with surgically resected, pathologically confirmed IPMNs were included in this study. Most cases *n* = 31 (67%) were classified as having low-grade IPMNs and were of the intestinal type, according to WHO 2019 criteria (Nagtegaal *et al*. 2020). The average age at diagnosis was 69.3 years, and the majority of patients were men, and self-identified as Non-Hispanic White (Table 1). A further breakdown of pathological features found among the 7 cases comprising the high-grade invasive cohort is found in Table 2; most (*n* = 5) were tubular (ductal). Tumor/plasma driver mutation concordance was analyzed for a subset of 15 patients for whom plasma was available.

**Table 1.** Demographic and clinicopathologic characteristics of the intraductal papillary mucinous neoplasm cohort (*n* = 46).

Characteristics	
Age at diagnosis, average	69.3 (median: 72; range: 48–82)
Sex, *N* (%)	
ȃMale	25 (54.3%)
ȃFemale	21 (45.7%)
Race/ethnicity, *N* (%)	
ȃNon-Hispanic White	39 (84.8%)
ȃNon-Hispanic Black	3 (6.5%)
ȃHispanic	4 (8.7%)
Cyst location, *N* (%)	
ȃHead	21 (45.7%)
ȃNeck, body, or tail	7 (15.2%)
ȃDiffuse	8 (17.4%)
ȃNA/not specified	10 (21.7%)
Cyst size, average (cm)	2.7 (median 2.5; range 0.7–5.0)
Epithelial subtype	
ȃPancreatobiliary	10 (21.7%)
ȃIntestinal	19 (41.3%)
ȃGastric	16 (34.7%)
ȃIOPN	1 (2.2%)
Histological grade	
ȃLow-grade	31 (67%)
ȃHigh-grade	9 (20%)
ȃHG-Invasive^a^	7 (13%)
Worrisome features	
ȃMain duct involvement	18 (39.1%)
ȃHRS: main duct ≥ 10 mm	7 (15.2%)
ȃHRS: obstructive jaundice	4 (8.7%)
ȃHRS: enhancing mural nodule ≥ 5 mm	8 (17.4%)
ȃSide branch IPMN	13 (28.3%)
Overall survival (mean, months)	
ȃMG	84.0
ȃHG	84.3
ȃHG-Invasive	64.1

^a^ȃAmong the high-grade cases, 7 (15%) had associated or concomitant invasive carcinoma.

**Table 2.** Characteristics of high-grade invasive histotypes (*n* = 7).

Histotype *N* (%)	
ȃTubular (Ductal)	5 (71.4%)
ȃColloid	1 (14.3%)
ȃUnknown	1 (14.3%)
Invasive IPMNs *N* (%)	
ȃIPMN with associated invasive carcinoma	3 (42.9%)^a^
ȃIPMN with a concomitant invasive carcinoma	3 (42.9%)^b^
ȃUnknown	1 (14.3%)

^a^ȃ1 intestinal, 1 gastric, and 1 pancreatobiliary. ^b^ȃAll intestinal.

### Mutational profile of IPMNs of the pancreas

When evaluating FFPE IPMN tissue (mean total read depth min = 212, max = 4,369, median = 510), *KRAS* driver mutations (G12D/R/V, G13D, and Q61H) were the most frequently detected mutations (37/46, 80.4%), followed by *GNAS* (R201H/C; 20/46, 43.5%) and truncating (protein) *RNF43* mutations (12/46, 26.0%). Other less common mutations included mutations in the genes *SMAD4* (truncating), *TP53* (both truncating and missense), and *APC* (truncating). A summary of the specific mutations detected in each sample along with parameters such as cyst size, epithelial subtype, and radiologic characteristics comprising high risk stigmata (HRS) and worrisome features is available in Supplementary Table 1.

**Fig. 1.**
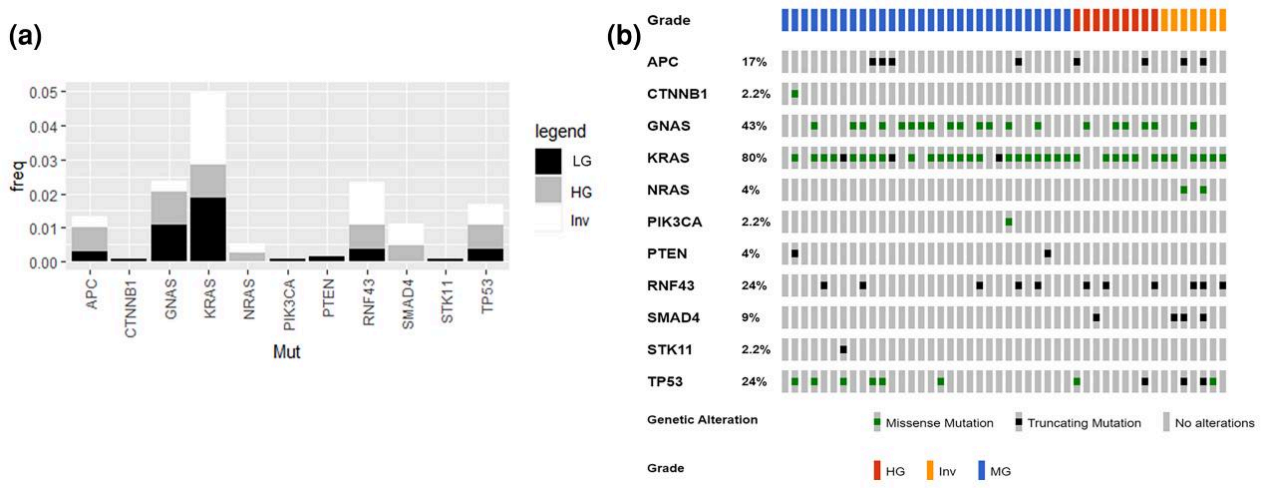
Frequencies of known driver mutations for each grade of IPMN. a) Frequency of mutations for each gene in each grade of IPMN was calculated and graphed. b) An oncoprint was generated using curated putative driver mutations for each sample. LG, low-grade; HG, high-grade; Inv, high-grade invasive.

**Fig. 2.**
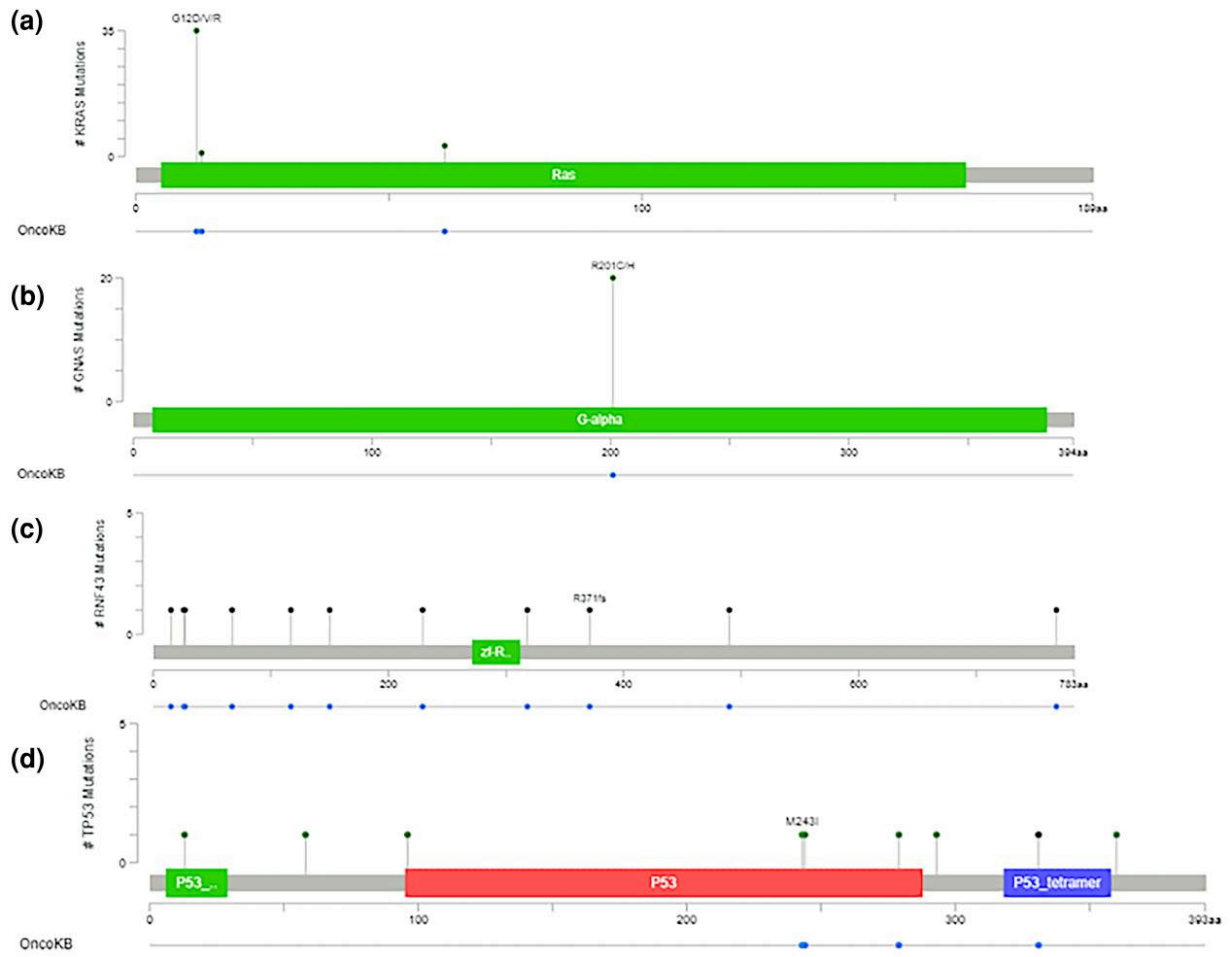
Mutational profile for top mutations in IPMNs. a) KRAS, b) GNAS, c) RNF43, and d) TP53 mutation identity/status was graphed using lollipop plots in cBioportal.

Frequencies for mutations for each grade of IPMN are shown in Fig. 1a and an oncoprint for all IPMN samples is shown in Fig. 1b. Interestingly, in this small cohort, we find a significant (*P*-value = 0.01) association between grade and *KRAS* mutations via the chi-square test. However, when a Cochrane–Armitage trend test was performed, mutation frequency did not trend with increasing or decreasing grade (trend test *P*-value = 0.6). We also observed a significant increase in the frequency of *RNF43* mutations from low-grade, to high-grade, to high-grade invasive IPMN (MG: 5/31; HG: 3/9; Inv: 4/7; *P*-value = 0.01) using a Cochrane–Armitage trend test. Lollipop plots for the 4 most common mutated genes (*KRAS*, *GNAS*, *RNF43,* and *TP53*) are shown in Fig. 2. No other driver mutations were detected in the panel of investigated genes based on our cutoff values.

In addition to using grade to group our cohort, we also conducted a retrospective classification of samples into the 3 main epithelial subtypes (intestinal, gastric, and pancreatobiliary). Using the epithelial subtype as a grouping system, we do not observe any significant differences in any of our profiled genes using a Cochrane–Armitage trend test.

Formal statistical analysis of our cohort by race/ethnicity was not performed due to the limited sample size and an uneven grade distribution. However, the exploratory analysis revealed that the only sample with a *CTNNB1* mutation derived from an individual of self-reported Hispanic/Latinx (H/L) background, *APC* driver mutations were found in samples from one African American and one H/L individual, and an *RNF43* mutation was identified in one H/L sample. Thus, 4 out of 7 non-NHW participants had IPMNs with mutations in the *Wnt* signaling pathway, compared to 16/39 for NHW (57% vs 41%; Papkoff *et al*. 1996; Koo *et al*. 2012; Giannakis *et al*. 2014; de Man & van Amerongen 2021; Sehgal *et al*. 2021).

### Mutational profile and concordance of mutations in resected tissue and paired plasma

The mutational profile of the resected tissue DNA (tDNA) and paired plasma DNA (ptDNA) was evaluated across the 15 IPMN cases for which plasma was available. Sample information may be found in Supplementary Table 1 and a summary of variant allele frequencies may be found in Supplementary Table 2. Prior to exclusion of known germline single nucleotide polymorphisms based on the 1,000 Genomes Project, we observed near perfect concordance (>99%) between plasma and FFPE samples. However, after exclusion of known germline variants, no concordant tumor driver mutations identified in the IPMN samples were detected in corresponding plasma despite adequate DNA concentrations for the assay (Supplementary Table 3). On the other hand, we did note very low allele frequencies (<2%) for one truncating *APC* mutation (p.Q2709X/p.Q2727X) in a confirmed low-grade case and 2 truncating *RNF43* mutations (possible driver mutations p.G456fs and p.Q414X) in 2 high-grade cases. However, these specific mutations were not detectable in the corresponding FFPE sample (Supplementary Table 1, note that each mutation call delineates different transcripts identified in RefSeq separated by a slash). For these reasons, we focused on mutational analysis of the IPMN tissues for further analysis.

## Discussion

Molecular characterization of IPMN tissue has the potential to inform medical management of premalignant pancreatic cysts, but there is an unmet need to develop noninvasive approaches for risk stratification. Findings from this preliminary study suggest that tumor DNA circulating in plasma using the metrics described herein will not serve as a proxy for cancer driver mutational status in determining IPMN dysplasia or risk stratification. QIAseq Human Cancer Panels have been shown to have high coverage, sensitivity, and concordance in detecting clinically relevant variants with minimal DNA input in other studies (Lam *et al*. 2020). Furthermore, Berger *et al*. demonstrated a concordance rate of ∼43% for GNAS driver mutations in a small cohort of IPMNs and paired liquid biopsy using digital droplet PCR, an arguably more sensitive methodology (Berger *et al*. 2016). However, in this study, we only observed possible truncating driver mutations in *RNF43* and *APC* among plasma samples at very low (<2%) allele frequency and these mutations were different than those observed in matched tumor samples. Others have reported that, while rare, clonal hematopoiesis may give rise to these “false positives” in other cancer types (Spoor *et al*. 2021). Hence, these very low frequency findings may be due to hematopoietic clonality or due to artifacts in this deep sequencing technique.

Although we did not conduct formal statistical analysis for race/ethnicity, we can report that a higher percentage of non-NHW displayed mutations in genes within the *Wnt* signaling pathway (57% vs 41%). These preliminary findings suggest that this pathway may be of increased importance in non-NHW pancreatic IPMN development or progression (Papkoff *et al*. 1996; Koo *et al*. 2012; Giannakis *et al*. 2014; de Man & van Amerongen 2021; Sehgal *et al*. 2021).

One interesting feature of our dataset is the fact that the frequency of *GNAS* R201H/R201C mutations is higher in low/high grade IPMNs vs invasive. *GNAS* mutations are generally a frequent occurrence in IPMNs and in other pancreatic lesions (Hosoda *et al*. 2015), although we find it less frequently in our cohort than others (43.5% vs 64% in other published studies; Hosoda *et al*. 2015). This may be due to small cohorts or differences in the populations under study.

Regarding our identified driver mutations, *KRAS* G12D/R/V, G13D and Q61H are all well-described activating mutations for GTPase signaling which lead to activation of multiple pro-survival and proliferation pathways (reviewed in Adjei 2001). The Gα_S_ (*GNAS*) R201 mutation functions similarly to inhibit its GTPase activity, thus blocking its return to an inactive state (Innamorati *et al*. 2018). On the other hand, our identified RNF43 mutations were varied and included truncating/splice site mutations p.W15X, p.27_27del, c.953-1G>A, p.R371X, p.V490fs, p.Q768X, p.G26fs, c.450+1G>A, p.G67fs and c.688-1G>A, all of which presumably inhibit RNF43's RING-type E3 ubiquitin ligase activity (Giannakis *et al*. 2014). *RNF43* functions are notable for the negative regulation of *Wnt* signaling (Koo *et al*. 2012). Hence, inactivating mutations for this protein may lead to dysregulation of cell polarity, migration, and invasion (Komiya and Habas 2008). We find that *RNF43* mutation frequency significantly increases with IPMN grade when classified under the pre-2019 WHO guidelines or under a two-tiered system. This finding suggests that *RNF43* mutation status may serve as a marker for IPMNs that warrant surgical intervention. Furthermore, *RNF43* mutational status is an indicator of sensitivity to *Wnt* pathway inhibitors (Jiang *et al*. 2013) and these mutations may thus be useful in predicting therapeutic response for pancreatic cancers which may arise from IPMN precursor lesions. Finally, putative driver mutations for *TP53* were denoted as any truncating mutation, or any mutation in the trans-activating domain, DNA binding domain or tetramerization domain under the assumption that these mutations would either inactivate or interfere with this protein's tumor suppressor functions (reviewed in Aubrey *et al*. 2016).

Some drawbacks of this study are a small sample size, lack of availability of adjacent normal tissue, the fact that NGS is less sensitive than ddPCR, the possibility of erroneous calls due to FFPE associated damage, and the fact that IPMNs may be spatially heterogeneous. Hence, mutational status for driver mutations may be present in the germline or may inaccurately represent the entirety of the specimen. Furthermore, since this study was conducted on archival tissue from as early as 2007, patients were originally classified under the pre-2019 WHO pathological guidelines. A retrospective re-classification was attempted using archival pathology reports. However, using only two grades of IPMN, significant differences were not observed between grades for any of the driver mutations identified in our cohort.

We did note a higher than reported mutation rate (see Supplementary Table 7) for the panel prior to curation. However, we do note that this panel sequences at a higher read depth (and therefore greater sensitivity) than others and that it is focused on cancer-related genes, explaining the high mutation rate. Additional confidence in the mutation calls comes from the application of molecular tag technology which not only eliminates duplicate reads but also leverages them to exclude sequencer or PCR errors.

Another limitation of the study arises from the fact that our samples were derived from blood. Others have demonstrated that sequencing of cfDNA from cyst fluid is a feasible method (Paziewska *et al*. 2020). However, we specifically did not use cyst fluid in our study since we wanted to focus on a less invasive method of sampling. Nevertheless, our results suggest that plasma may not be as helpful a sample type as cyst fluid for driver mutation detection.

### Conclusions

We conclude that, in our hands, the QIAseq custom cancer panel is not a reliable method to detect potential driver mutations in liquid biopsies from IPMN patients. Indeed, the findings of Berger *et al.* (2016) suggest that ddPCR is a more reliable, but still imperfect method for concordance analysis using liquid biopsies from patients with IPMNs. More sensitive methodologies and alternate classes of blood-based biomarkers such as miRNAs (Permuth-Wey *et al*. 2015) warrant further investigation to guide the medical management for the growing number of individuals diagnosed with IPMNs each year.

## Supplementary Material

jkac314_Supplementary_Data

## Data Availability

Supplementary Table 1 contains detailed descriptions of all patient demographics and a summary of observed driver mutations. Supplementary Table 2 contains information regarding the frequency of alternative alleles. Supplementary Table 3 and Supplementary Table 4 contain data regarding cfDNA and FFPE extracted DNA purity and amount. Supplementary Tables 5 and 6 contain sequence summary statistics for FFPE and plasma samples. A summary of all mutations detected via SmCounter is contained in Supplementary Table 7 and a list of all custom gene sequenced is contained in Supplementary File 1. Raw sequencing files can be found under dbGAP ID (phs003043: https://www.ncbi.nlm.nih.gov/projects/gap/cgi-bin/study.cgi?study_id=phs003043.v1.p1). Supplemental material available at G3 online.

## Notes

*Communicating editor: J. Prendergast*
